# Cross-linguistic effects of pre-reading prediction, cue encoding, and retrieval interval on metacomprehension monitoring accuracy

**DOI:** 10.3389/fpsyg.2026.1774952

**Published:** 2026-04-21

**Authors:** Fei Xu, Yin Guo, Mingju Yang, Jinxiu Chen

**Affiliations:** School of Humanities and Foreign Languages, Qingdao University of Technology, Qingdao, China

**Keywords:** cross-linguistic transfer, interval for clue encoding and retrieval, language proficiency, metacomprehension monitoring accuracy, pre-reading prediction

## Abstract

Recent studies show self-explanation and predictive inference strategies can improve metacomprehension monitoring, but the roles of pre-reading prediction and cross-linguistic transfer remain under-examined. This study used a 2 (language type: Chinese vs. English) × 2 (predictive condition: predictive vs. non-predictive) × 2 (interval type: interval vs. no-interval) × 2 (language proficiency: Chinese vs. English scores) mixed design, with immediate judgments and segmented delayed measures, to test effects of pre-reading prediction and encoding-retrieval interval and their transfer across languages. Key findings of the current research include (1) no-interval conditions yielded higher monitoring accuracy than interval conditions; (2) the pre-reading prediction condition yielded lower monitoring accuracy for expository texts, likely due to limited prior knowledge and the specific demands of the pre-reading task; (3) L1 (Chinese) metacomprehension was transferred to L2 (English) contexts, while foreign-language proficiency did not mediate transfer. These results clarify how encoding-retrieval intervals affect metacomprehension accuracy in reading in Chinese, delimit the conditions under which pre-reading prediction may be counterproductive, and provide empirical evidence for cross-linguistic transfer of metacomprehension processes in bilingual settings. Empirically, the findings suggest that pre-reading prediction training should be tailored to text genre and readers’ background knowledge, and that strengthening L1 metacognitive skills can facilitate L2 reading.

## 1 Introduction

Metacomprehension refers to readers’ monitoring of their understanding and the regulation of subsequent reading strategies, is a central construct in cognitive science and educational psychology (Chen and Chang, 2009). Accurate metacomprehension monitoring is widely recognized as a prerequisite for effective strategy regulation, yet robust evidence shows that learners across levels, including university students, frequently exhibit limited monitoring accuracy (Dunlosky and Lipko, 2007; Lin and Zabrucky, 1998). Prinz et al. (2020) found through a meta-analysis that relative metacomprehension accuracy is modest and shaped by learner-, text-, judgment-, and test-related factors. Instruction should therefore foster monitoring via practice texts, immediate predictions, and suitable test formats. Addressing this limitation therefore remains both a theoretical priority and an instructional imperative.

A growing body of intervention research has identified tactics that can improve monitoring accuracy. Notably, self-explanation and pre-reading prediction have produced reliable benefits in various learning contexts (Griffin et al., 2008; Thiede et al., 2009). However, recent research shows that such benefits may be limited for expository texts when readers lack sufficient prior knowledge, and that a study goal akin to prediction instruction can even be counterproductive under those circumstances (Mengelkamp et al., 2025). Theoretically, these strategies are thought to operate by activating and elaborating prior knowledge structures (schema theory; Rumelhart, 1980) and by supporting coherent situation model construction during comprehension (Kintsch, 1988). Empirically, however, several substantive questions remain unresolved.

First, although prediction overlaps conceptually with self-explanation, pre-reading prediction has seldom been treated as an independent instructional variable; its direct effects and boundary conditions (e.g., dependence on prior knowledge or text genre) are underexamined. Second, temporal factors, specifically the interval between cue encoding and retrieval, have been shown to influence metacomprehension in some foreign language studies (Yan et al., 2015), but comparative evidence is sparse, particularly in Chinese reading contexts. Moreover, individual differences in verbal working memory may moderate the effectiveness of such interval effects, as readers with higher working memory capacity tend to benefit more from strategy instruction on lengthy expository texts (Bagri and Abrar, 2024). Third, much of the existing literature is monolingual, leaving open whether strategy and temporal effects generalize across languages and whether metacomprehension monitoring skills transfer from learners’ L1 to their L2. Finally, the potential mediating role of target language proficiency in any cross-linguistic transfer has received little empirical scrutiny.

Representative work has thus established important foundations (e.g., Chen and Chang, 2009 on construct centrality; Lin and Zabrucky, 1998, and Dunlosky and Lipko, 2007 on accuracy shortfalls; Griffin et al., 2008, and Thiede et al., 2009 on strategy effects; Yan et al., 2015 on interval influences), but the field lacks systematic experimental tests that integrate strategy, temporal spacing, language type, and proficiency within a cross-linguistic framework. Additional unresolved issues include how genre and prior knowledge moderate strategy effectiveness (e.g., why prediction may fail for expository texts), whether immediate versus segmented delay measures capture different facets of monitoring, and how instruction might differentially support metacognitive transfer across languages.

Recent evidence from metacognitive training studies suggests that metacognitive regulation skills can transfer across qualitatively different types of learning strategies (Wirth et al., 2025) and that even simple procedural interventions can improve predictive metacognitive sensitivity without being tied to a specific language (Proust et al., 2025). Moreover, offline self-report measure of proficiency often fails to capture the same constructs as online monitoring accuracy (Seban et al., 2025). And high prior knowledge can paradoxically reduce monitoring accuracy (Witherby et al., 2023). Resolving these gaps is critical for advancing theoretical models of metacomprehension in bilingual contexts, as current frameworks remain largely grounded in monolingual paradigms that do not account for cross-linguistic dynamics. Moreover, clarifying how instructional strategies such as prediction interact with temporal factors and language proficiency has direct pedagogical implications, informing the design of evidence-based reading interventions for L2 learners.

The present study responds to these gaps. Focusing on university learners whose L1 is Chinese and whose L2 is English, it addressed two primary research questions: (1) From a cross-linguistic perspective, do pre-reading prediction and the encoding-retrieval interval influence metacomprehension monitoring accuracy, and do these factors interact? (2) Is there cross-linguistic transfer of metacomprehension monitoring ability between Chinese (L1) and English (L2), and if so, is that transfer mediated by target language proficiency? Guided by schema theory (Rumelhart, 1980) and the situation model hypothesis (Kintsch, 1988), we presented two hypotheses. Firstly, we hypothesize that prediction facilitates monitoring by activating and elaborating relevant schemas and supporting situation model construction, but that its effectiveness will depend on prior knowledge and text genre. Secondly, we hypothesize that time interval will modulate cue availability and thus monitoring accuracy, and that L1 monitoring skills may transfer to L2 contexts irrespective of L2 proficiency level.

## 2 Theoretical foundations and key constructs

### 2.1 Reading strategies and pre-reading prediction

Reading is an active, hypothesis-driven process in which readers generate predictions, test hypotheses, and revise mental representations (Goodman, 1967; Yu, 2006). Prediction has long been recognized as a higher-level skill: readers use titles, textual cues, and prior knowledge to anticipate forthcoming content and discourse structure (Duan, 2004; Nuttall, 2002).

Pre-reading prediction is defined as a deliberate, instructionally cued generation of expectations about upcoming text content before full text exposure. Theoretically grounded in schema theory (Rumelhart, 1980) and the situation model hypothesis (Kintsch, 1988), it follows Matsumoto’s (2011) structuring of prediction activities prior to reading to activate schemata. Activated schemata provide background frameworks that readers draw upon to generate and refine predictions, and successful prediction contributes to coherent situation models that integrate text-based information with world knowledge (Kintsch, 1988; Wang and Li, 2014). Empirical work shows that situation model construction and retrieval enhance metacomprehension monitoring (Anderson and Thiede, 2008; Chen and Chang, 2009; Rawson and Kintsch, 2005).

Pre-reading prediction is distinct from three related constructs: predictive inferences, which occur automatically during online comprehension (Cain and Oakhill, 1999; Murphy et al., 1993); predictive strategies, which involve continuous hypothesis testing throughout reading (Goodman, 1967); and self-explanation, which focuses on explaining explicit text content to integrate with prior knowledge rather than anticipating upcoming information (Griffin et al., 2008). Despite these theoretical links, pre-reading prediction itself has been under-tested as an independent instructional variable: its direct effects, boundary conditions (e.g., text genre, prior knowledge), and cross-linguistic generalizability remain insufficiently explored.

No research directly examines prior knowledge × pre-reading prediction interactions in L2 reading, despite prediction’s theoretical centrality (Goodman, 1967). Witherby et al. (2023) found in L1 that prior knowledge positively predicted learning but negatively predicted predictive accuracy, namely, high-knowledge students relied on heuristic familiarity over diagnostic cues. For L2 reading, high L1 topic knowledge may thus lead learners to overestimate L2 comprehension, trusting familiarity cues that become less diagnostic under language processing demands. Soto et al. (2019) further showed that distinct metacognitive components support inferential versus text-based comprehension, a distinction critical for L2 readers who must flexibly deploy strategies based on proficiency and text demands.

### 2.2 Metacomprehension monitoring: constructs and measurement

Metacomprehension, the monitoring and regulation of comprehension, derives from metacognitive theory (Flavell, 1979) and has been operationalized as readers’ explicit judgments of their understanding (Brown, 1982; Matlin, 2005). Research typically distinguishes monitoring (diagnostic judgments) from regulation (strategy adjustments), with monitoring accuracy being treated as foundational for effective regulation (Chen and Wen, 2010; Koriat, 1997).

Two principal metrics have been used. Absolute accuracy captures calibration between judged and actual performance (e.g., Brier scores, calibration curves), revealing over- or underestimation biases. Relative accuracy assesses the degree to which confidence judgments discriminate between better-understood and worse-understood items, commonly indexed by Goodman–Kruskal gamma or Pearson correlations (Maki et al., 2005; Nelson et al., 2004). Relative accuracy is widely used in metacomprehension research because it reflects discriminatory monitoring relevant to study-and-test decisions; it is therefore the primary outcome in the present study.

Despite robust interest, monitoring accuracy is typically low: mean gamma correlations cluster near 0.25–0.27 across studies (Dunlosky and Lipko, 2007; Lin and Zabrucky, 1998), indicating persistent constraints on learners’ ability to judge comprehension reliably.

### 2.3 Temporal factors and the transfer-appropriate monitoring hypothesis

A key advance in metacomprehension research is the transfer-appropriate monitoring hypothesis: monitoring accuracy depends on congruence between cue encoding and the demands of the criterion test (Chen and Li, 2008; Thiede et al., 2003, 2005). Encoding and retrieval conditions shape which cues (e.g., processing fluency versus retrieval fluency) are available at judgment time; immediate judgments tend to rely on short-term fluency cues, whereas delayed or segmented measures shift judgments toward retrieval-based cues (Koriat and Ma’ayan, 2005; Rawson and Dunlosky, 2002). Yan et al. (2015) compared instant versus segmented delay protocols and found that immediate conditions typically yielded higher monitoring accuracy, with some proficiency-dependent patterns: higher-proficiency readers benefited more from immediate measures, while lower-proficiency readers sometimes performed better on delayed measures. However, most of this evidence derives from alphabetic languages (often English), and comparative work in Chinese reading and cross-linguistic settings is scarce.

This gap matters because the logographic nature of Chinese may affect how readers encode and retrieve text information, altering cue availability for metacomprehension. In alphabetic languages, higher-proficiency readers benefit from immediate measures, whereas lower-proficiency readers sometimes perform better on delayed measures. Chinese readers, even with high proficiency, may process language with less automaticity, potentially shifting how interval effects operate. Thus, findings from alphabetic languages may not generalize directly to Chinese reading, underscoring the need for cross-linguistic research.

### 2.4 Pre-reading prediction and metacomprehension

Pre-reading prediction in this study is operationalized as an instructional variable comprising two components derived from Yang and Qin (2007): activation of content schemata (recalling prior knowledge to guess content based on the title) and activation of formal schemata (formulating questions answerable in the article, speculating about structural arrangement, and guessing content development based on text structure). Unlike predictive inferences, which occur automatically during online comprehension (Cain and Oakhill, 1999; Murphy et al., 1993), pre-reading prediction is deliberately prompted before text encounter. It also differs from self-explanation, which focuses on explaining explicit text content rather than anticipating upcoming information (Griffin et al., 2008).

Experimental research indicates that when readers base judgments on situation-model cues rather than surface or text-based cues, metacomprehension accuracy improves (Thiede et al., 2009). Theoretical accounts such as the interference-level framework of Dunlosky et al. posit that reasoning at the situation-model level helps detect logical inconsistencies and thus refines judgments (Dunlosky et al., 2002). Intervention studies show that reasoning practice and self-explanation training can raise metacognitive accuracy by strengthening situation models (Griffin et al., 2008; McNamara, 2004; Moss and Schunn, 2015). However, the effectiveness of such interventions may depend on readers’ verbal working memory capacity and learners with higher working memory tend to benefit more from strategy instruction on complex expository texts (Bagri and Abrar, 2024).

Despite these established links, direct empirical tests isolating pre-reading prediction as a strategy that influences metacomprehension monitoring remain sparse. Whether explicit prediction instructions improve, impair, or interact with temporal cue-availability remains unresolved, particularly for expository genres where prior knowledge may be insufficient to generate accurate expectations.

### 2.5 Cross-linguistic transfer and the mediating role of proficiency

Most metacomprehension research has been monolingual, leaving open whether monitoring skills transfer across languages. Two competing perspectives exist. The Reading Universal hypothesis suggests that reading skills, including metacognitive monitoring, transfer broadly across languages (Coady, 1979). In contrast, the Linguistic Threshold hypothesis argues that transfer depends on sufficient target-language proficiency (Pichette and Segalowitz, 2003). Empirical evidence is limited and often restricted to linguistically similar languages or immersion contexts (e.g., Morrison, 2004). In bilingual contexts where L1 and L2 differ typologically (e.g., Chinese-English), factors such as lexical knowledge, syntactic habits, and culturally embedded schemata may differentially constrain prediction and situation-modeling processes (Pan, 2005). Thus, whether native-language metacognitive monitoring supports L2 monitoring independently of L2 proficiency, or whether proficiency mediates transfer, is an open empirical question with direct curricular and pedagogical implications. Recent research outside the bilingual domain provides evidence that metacognitive regulation skills can be transferred across different types of learning strategies. This is a phenomenon termed as far transfer of metacognitive regulation (Wirth et al., 2025). Moreover, procedural training that simply asks learners to predict, evaluate and compare their performance has been evidenced to improve metacognitive sensitivity without being confined within a specific language or task (Proust et al., 2025). These findings suggest that domain-general metacognitive skills may be more basic than language-specific competencies to explain cross-linguistic transfer.

Direct research on Chinese-English bilinguals is limited. Ackerman et al. (2023) found that Chinese participants demonstrated distinctive effort regulation reflecting Confucian values investing more time, achieving higher success, but showing lower efficiency suggesting cultural factors moderate metacognitive engagement.

### 2.6 Metacomprehension research with expository texts: a systematic review

Expository texts have been a central focus of metacomprehension research precisely because they lack the familiar story structures of narratives, making situation model construction more effortful and metacognitive monitoring more essential (Weaver and Bryant, 1995). A key finding in this literature is that metacomprehension accuracy is typically lower for expository than for narrative texts, and this gap is often attributed to the cognitive demands of expository texts including lower coherence, domain-specific terminology, and greater reliance on prior knowledge which require readers to engage in more deliberate inferencing and self-assessment. Early work by Weaver and Bryant (1995) established a nonlinear relationship between text difficulty and metamemory accuracy for expository texts (the “optimum effort hypothesis”), a pattern that underscores how text complexity interacts with monitoring processes. Subsequent research has further clarified the role of metacognitive knowledge: Lin et al. (2000) found that metacomprehension knowledge better predicts comprehension for expository than for narrative texts, suggesting that readers’ explicit awareness of strategies becomes more critical when texts impose greater cognitive demands. Soto et al. (2019) extended this by showing that different metacognitive components serve distinct functions in expository reading: planning and evaluation predict inferential comprehension, whereas monitoring predicts text-based comprehension.

Importantly, research on expository texts has also revealed methodological considerations for intervention design. Gordon and Pearson (1983) showed that strategy training benefits only higher-ability readers, suggesting a minimal proficiency threshold for intervention effectiveness, a finding that is particularly critical for expository text processing, where cognitive demands are elevated. Collectively, this body of research establishes that expository texts present unique challenges for metacomprehension, but it also leaves open questions about how instructional strategies such as pre-reading prediction interact with text characteristics, prior knowledge, and reader proficiency, gaps the present study addresses.

### 2.7 Interaction between prior knowledge and predictive strategy application in L2 reading

Schema theory posits that prior knowledge structures guide comprehension, generate predictions, and fill gaps in the text (Rumelhart, 1980). The effectiveness of pre-reading prediction therefore depends critically on the quality and availability of that prior knowledge. In L1 reading, Witherby et al. (2023) found a nuanced relationship: prior knowledge positively predicted learning outcomes but negatively predicted predictive accuracy, a pattern attributed to high-knowledge readers’ reliance on heuristic familiarity cues rather than diagnostic text-based cues. This finding highlights a potential boundary condition: prediction effectiveness may be moderated by the degree to which readers’ prior knowledge is congruent with the text’s content.

In L2 reading, this interaction becomes more complex due to additional constraints of language proficiency. While L2 readers may possess relevant domain knowledge in their native language, their ability to activate and apply that knowledge during L2 reading may be compromised by limited vocabulary, unfamiliar syntax, or slower processing speed. This dissociation between knowledge availability and application is critical for understanding how prediction functions in L2 contexts. Soto et al. (2019) provided indirect evidence that inferential comprehension, the type most essential for generating accurate predictions, is best predicted by planning and evaluation skills, which themselves depend on readers’ ability to integrate text information with prior knowledge. Furthermore, offline self-report measures of proficiency tend to have a poor relationship with online monitoring accuracy, for judgment-based online measures are more strongly related with actual performance (Seban et al., 2025). Also, high prior knowledge can paradoxically reduce predictive monitoring accuracy because knowledgeable learners tend to remember items they thought they would forget (Witherby et al., 2023). These findings highlight that domain-general metacognitive skills and the cues used during monitoring might be more essential than the sheer amount of prior knowledeg or proficiency.

Direct empirical investigations of the prior knowledge × pre-reading prediction interaction in L2 reading remain scarce. The present study directly tests these possibilities by comparing the effects of pre-reading prediction across Chinese (L1) and English (L2) reading contexts, with L2 proficiency included as a continuous variable to examine its moderating role.

## 3 Method

The present study employed a 2 (language type: Chinese vs. English) × 2 (encoding–retrieval interval: immediate judgment vs. segmented delay judgment) × 2 (predictive condition: predictive group vs. non-predictive group) mixed experimental design, with language type and encoding-retrieval interval as within-participants factors and pre-reading prediction as a between-participants factor. Language proficiency was indexed by participants’ College Entrance Examination scores in Chinese and English and was included as a continuous covariate. In the present study, the Gaokao English score was used as a broad measure of general English proficiency rather than as a fine-grained measure of language ability. Because it derives from a standardized, externally administered examination taken by all participants under comparable conditions prior to university entry, it provides a common and objective background index of overall English attainment for participants. In the current design, this measure was used to control for between-participant differences in general L2 proficiency, rather than to assess domain-specific reading ability or metacomprehension directly. The dependent variable was metacomprehension monitoring accuracy, operationalized as the relative accuracy (Goodman-Kruskal gamma correlation) between participants’ comprehension judgments and their actual test performance on expository texts. The effects of these factors and their interactions on monitoring accuracy were examined using stepwise regression analysis. The experiment was carried out in a quiet classroom setting at the participating university.

### 3.1 Participants

The participants of this study were randomly selected from 70 first-year undergraduate students majoring in English at a university, of whom 59 were female and 11 were male (*M*_age_ = 18.62, *SD* = 0.52). Participants’ language proficiency was measured using their College Entrance Examination (Gaokao) scores for Chinese and English. The Gaokao is a standardized, high-stakes selection test for tertiary education in China, administered under identical conditions nationwide and centrally graded to ensure comparability (Shi, 2025; Zhao et al., 2022). Its English subtest assesses a broad range of language competencies including reading comprehension, vocabulary, grammar, and writing aligned with national curriculum standards, and has demonstrated satisfactory reliability and validity in predicting subsequent college academic achievement (Shi, 2025; Zhao et al., 2022). While Gaokao scores reflect general academic English proficiency rather than reading-specific skills, they serve as a valid proxy for overall language ability, which is sufficient for covariate adjustment.

The use of Gaokao scores as covariates is well established in educational and psychological research. Prior studies have employed them to control for baseline academic ability in analyses of college performance (Li and Xu, 2022), and student development (Liu et al., 2018). Following these precedents, we entered Gaokao scores as covariates to control for between-participant variation in general language proficiency, thereby isolating the effects of the experimental manipulations on metacomprehension monitoring accuracy.

### 3.2 Materials

#### 3.2.1 Criterion test materials and metacomprehension monitoring estimation form

1. Criterion test material

In this study, eight English and eight Chinese expository texts were selected as experimental materials, with topics in the fields of education, culture, society, science and technology, and art etc. The length of the English short texts was controlled to be between 300 and 400 words, and the length of the Chinese short texts was controlled to be about 850 to 1,100 words. Five multiple-choice questions of comparable difficulty were designed after each reading material to examine the test takers’ comprehension and monitoring ability of the short texts, and the test was standardized into a four-choice multiple-choice test, in which the ratio of inference questions to detail questions was 4 to 1. Detail questions were designed according to a specific sentence or paragraph to examine the text-based representation of chapter comprehension, while inference questions required test takers to reason based on their background knowledge to answer the questions correctly. Examples of detail and inference questions for a sample text are provided in Appendix III. Reasoning questions are questions that require the test taker to make inferences based on background knowledge to answer correctly, and they mainly examine the test taker’s understanding of the content of the text.

A norming study was conducted with 30 participants drawn from the same population as the main experiment. Each participant rated the eight Chinese and eight English expository texts on two 7-point Likert scales: topic familiarity (1 = completely unfamiliar, 7 = very familiar) and text difficulty (1 = very easy, 7 = very difficult). Texts were presented in random order via Wenjuanxing, a Chinese online survey platform widely used in academic research for questionnaire administration and data collection (Changsha Ranxing Information Technology Co., Ltd.). All data collected through the platform were exported and subsequently analyzed using SPSS 26.0.

Descriptive statistics showed that all texts received mean familiarity ratings between 3.95 and 4.20 (*SD* = 0.86–0.97) and mean difficulty ratings between 3.95 and 4.20 (*SD* = 0.86–0.95). No text had a mean rating below 3.5 or above 4.5, and all standard deviations were below 1.0, indicating that none of the texts were perceived as extreme outliers in terms of familiarity or difficulty.

To evaluate whether the texts within each language group were perceived as internally consistent, Cronbach’s *α* coefficients were calculated separately for the eight Chinese texts and the eight English texts. For topic familiarity, Cronbach’s α was 0.87 for Chinese texts and 0.86 for English texts. For text difficulty, Cronbach’s α was 0.85 for Chinese texts and 0.84 for English texts. All *α* values exceeded 0.80, indicating high internal consistency. These results suggest that participants rated the texts within each language group as comparable in both familiarity and difficulty, supporting the appropriateness of the text sets for cross-linguistic comparison.

1. Metacomprehension monitoring assessment form: participants were asked to rate their comprehension on a scale of 1–5 (1 = not at all, 5 = completely) after reading. An example is shown in Appendix IV.

#### 3.2.2 Setup of the prediction pretest portion of the experimental design

In this study, to fully activate readers’ content schemata and formal schemata and to encourage readers to actively use pre-reading prediction, we divided all participants into predictive and non-predictive groups. Specifically, we designed topic prediction questions for article titles and content prediction questions for article structure and content. All questions were in the form of four-choice multiple-choice questions, with one title prediction question and one content prediction question for each article. The predictive group was provided with the title and part of the article before reading the complete article and was then asked to complete the corresponding prediction questions after reading the article. On the other hand, the non-predictive group will only be provided with titles and parts of the article.

This design created two different pre-reading conditions: a prediction-based pre-reading condition, in which participants actively generated predictions from partial textual cues, and a non-predictive preview condition, in which participants previewed comparable cues without generating predictions. The comparison was intended to examine how these different pre-reading activities were associated with subsequent metacomprehension monitoring accuracy.

1. Topic prediction questions

Based on the first two aspects of the predicting strategy defined by Yang and Qin (2007), we designed topic prediction questions. These questions were designed to activate the reader’s content schemata, i.e., the sum of the reader’s original knowledge about the content of the article. The topic prediction questions required participants to recall knowledge that might be relevant to the article after seeing the article’s title and to guess the content or point of view of the article based on the article’s title or the given schema. An example is shown in Appendix I.

1. Structural content prediction questions

Based on the latter three aspects of the predicting strategy defined by Yang and Qin (2007), we designed structural content schemata prediction questions designed to activate the reader’s formal schemata, in other words, the reader’s knowledge system about the organization and composition of the text. These questions required participants to formulate questions that might be answered in the article based on the article’s title, to speculate about the article’s structural arrangement, and to guess the development of the article’s content or the author’s point of view based on the article’s structure. An example is shown in Appendix II.

With this prediction pretest design, both groups were exposed to the article title before full-text reading, but the predictive group was additionally required to complete explicit prediction questions. More specifically, in each trial both groups first viewed the title for 1 min; the predictive group then received 4 min to inspect the title and partial text and complete the prediction questions, whereas the non-predictive group received 3 min to inspect the title and partial text without answering prediction questions. Thus, the total pre-reading time differed by 1 min (5 vs. 4 min) because the predictive condition involved an additional response requirement, not because participants were given extra full-text reading time. This procedural choice was intended to allow completion of the prediction task under manageable time pressure. At the same time, because the two conditions differed in both instructional demand and pre-reading task duration, the resulting contrast should be interpreted as a comparison between two pre-reading conditions rather than as a pure isolation of prediction instruction.

### 3.3 Experimental procedures

The experiment was divided into two phases: the first phase being an instant judgment metacomprehension test, and the second phase being a segmented delay metacomprehension test. One week after the completion of the first phase of the experiment, the second phase of the experiment was conducted on the same all the participants without their knowledge. Both experiments were conducted in a classroom in a quiet and comfortable environment. Both experiments were completed through paper and pencil tests, and each experiment took approximately 80 min. The experiments were divided into two groups according to the number of participants: predictive group and a non-predictive group.

To prevent potential practice effects and to ensure that performance in the second phase reflected genuine metacomprehension processes rather than memory of specific test content, participants were instructed not to discuss the experiment with others, not to revisit any of the reading materials or test questions, and not to engage in any additional English or Chinese reading comprehension practice that might systematically influence their performance during the one-week interval. No formal checks were administered beyond these verbal instructions, as the aim was to maintain naturalistic conditions while minimizing explicit demand characteristics. Participants were not informed in advance that a second phase would occur; they were told only that the experiment had concluded after Phase 1.

#### 3.3.1 Phase 1: instant judgment (non-interval) metacomprehension test

1. Predictive group

For the English test, the experiment was conducted after familiarizing the participants with the entire experimental procedure. Before reading, the title of the first article and the corresponding title prediction questions were first given to the participants, who were asked to read and complete the corresponding questions within 1 min, and were uniformly retrieved after the time, then the title and part of the article as well as the corresponding content structure prediction questions were given to the participants, who were asked to read and complete the corresponding questions within 4 min, and uniformly retrieved after the time, and then the first short article was given to the participants in its entirety, and then the participants were asked to read it within 5 min and uniformly retrieved it when the time was up. Next, the participants were immediately given the metacomprehension monitoring assessment form and asked to rate their comprehension of the general idea of the text on a scale of 1–5 (1 = not at all, 5 = completely). The assessment form was retrieved after 1 min, and then the reading test questions were distributed to the participants, who were asked to complete all the four-choice multiple-choice questions within 5 min. This was repeated until the last short-text test question was completed.

The predictive Chinese test is as above.

1. Non-predictive group.

For the English test, the experiment was conducted after the participants were familiarized with the whole experimental procedure. Before reading, the title of the first article was first given to the participants, who were asked to read it within 1 min, after which the material was uniformly retrieved. Next, the title and part of the article were given to the participants. They were asked to read it within 3 min, after which the materials were uniformly retrieved. Then, the first short article was given to the participants in its entirety. They were asked to read it within 5 min and uniformly retrieve it when the time was up. Immediately thereafter, the participants were given the metacomprehension monitoring assessment form and asked to rate their understanding of the general idea of the text on a scale of 1–5 (1 = not at all, 5 = completely). The assessment form was returned after 1 min. Finally, the reading test questions were distributed to the participants, who were asked to complete all four-choice multiple-choice questions within 5 min. This was repeated until the last short-text test question was completed.

The non-predictive Chinese test is as above.

Then, without the participants’ knowledge, Phase 2 (Segmented Delay) was conducted one week later.

#### 3.3.2 Phase 2: segmented delay (interval) metacomprehension test

1. Predictive group

For the English test, the experiment was conducted after familiarizing the participants with the whole experimental procedure. Before reading, the title of the first article and the corresponding title prediction questions were first given to the participants. They were asked to read and complete the corresponding questions within 1 min, after which the materials were uniformly retrieved. Next, the title and part of the article, along with the corresponding content structure prediction questions were given to the participants. They were asked to read and complete these questions within 4 min, after which the materials were uniformly retrieved. Then, the first short article was then given to the subject in its entirety, and they were asked to read it in 5 min, after which the materials were uniformly retrieved. Immediately thereafter, the participants were given the metacomprehension monitoring assessment form and asked to rate their understanding of the general idea of the article on a scale of 1–5 (1 = not at all, 5 = completely). The assessment form was uniformly withdrawn after 1 min. Finally, the reading test questions were distributed to participants, who were asked to complete all four multiple choice questions within 5 min. This sequence will continue until the last article is completed. The order in which the assessed articles and reading test articles are presented is the same as the order in which the participants read the articles.

The predictive Chinese test is as above.

1. Non-predictive group

For the English test, the experiment was conducted after familiarizing the participants with the entire experimental procedure. Before reading, the title of the first article was first sent to the participants, who were asked to read it within 1 min and uniformly retrieved after the time, then the title and part of the article were sent to the participants, who were asked to read it within 3 min and uniformly retrieved after the time. Next, the first short article was sent to the participants in its entirety, along with the after which the participants were asked to read it within 5 min and uniformly retrieved after the time, and so on until the last article was read. The first article was then given to the subject in its entirety. Participants were then immediately given the metacomprehension monitoring assessment form for the first article and asked to first rate their understanding of the general idea of the article on a scale of 1–5 (1 = not at all, 5 = completely), and the assessment forms were returned after 1 min, and so on until the last article was completed. The first reading test was given to the participants and they were asked to complete all four multiple choice questions within 5 min. This will continue until the last article was completed. The order in which the assessed articles and reading test articles are presented is the same as the order in which the participants read the articles.

The non-predictive Chinese test is as above.

### 3.4 Statistical analysis

In this study, a mixed design of 2 (language type: Chinese/English) × 2 (interval type: interval/non-interval) × 2 (predictive condition: predictive/non-predictive) × 2 (language proficiency: Chinese scores/English scores) was used, and the effects of these factors on metacomprehension monitoring accuracy were investigated by stepwise regression analysis.

Through data collection and screening, the data of 11 participants who did not complete the experimental materials or omitted important information were excluded, leaving 59 participants (52 females and 7 males) for analysis. Their College Entrance Examination scores for Chinese (*M* = 115.60, *SD* = 7.42) and English (*M* = 121.70, *SD* = 7.61) were entered as covariates indexing general L1 and L2 proficiency. Then, the data were statistically analyzed using R4.5 statistical software.

Linear mixed-effects models were fitted using the lme4 package (Bates et al., 2015) in R 4.5 (R Core Team, 2022) to examine the effects of prediction condition (predictive/non-predictive), interval type (interval/non-interval), language proficiency as indexed by College Examination scores in Chinese and English, language type (Chinese/English), and their interactions on metacomprehension monitoring accuracy. In the models, subject was included as a random effect, and significance tests were performed using Satterthwaite’s degrees of freedom approximation method.

The analysis was conducted using a stepwise regression strategy, starting from the base model and adding main effects and interaction terms sequentially. Analysis of variance (ANOVA) revealed a significant contribution of interval type in the main effects model (*F*(1, 233) = 31.34, *p* < 0.001); the interaction of language type and interval type significantly improved the model fit in a second-order interaction model comparison, *F*(1, 229) = 10.01, *p* = 0.002; and the third-order interaction model also showed a borderline significant improvement (*F*(3, 226) = 2.70, *p* = 0.05). The above suggests that the third-order interaction model used in this study explains the variance in the data well (*R*^2^ = 0.21, *F*(9, 226) = 6.74, *p* < 0.001) when the interaction of all three factors (language type, interval type, and predictive type) is taken into account.

A thorough diagnostic analysis of the third-order interaction model revealed that the tests for multicollinearity showed that all VIF values were within an acceptable range: the VIF values for the main effects terms were distributed between 1.01 and 4.03 (Language: 3.19, Interval Type: 3.19, Predictive: 4.03, Chinese_score: 1.04, English_score: 1.01), indicating no multicollinearity problem for the main effects. The VIF values for the interaction effect terms were slightly higher but did not exceed 6 (Language × Interval Type: 4.78, Language × Predictive: 5.19, Interval Type × Predictive: 5.19, third-order interaction: 5.78) indicating no multicollinearity problems (Belsley et al., 1980).

The third-order interaction model chosen for this study was able to better account for the variance in the data, indicating that the interaction of the three factors of language type, interval type, and predictive type had a significant effect on the accuracy of metacognition judgments. After diagnostic analysis, although the VIF values of some of the interaction effect terms were slightly higher in the multiple covariance test part, they were all within the acceptable range, indicating that there was no serious problem of multiple covariance among the variables of the model, and the parameter estimation of the model was reliable. Therefore, further analysis and interpretation can be carried out based on the model, and the conclusions drawn have a high degree of confidence.

Specifically, Final model chosen is: lm(formula = GammaCorrelations ~ Language * IntervalType * Predictive + Chinese_score + English_score, data = df_long).

## 4 Results

All categorical variables were effect-coded (sum-coded) using a (1, −1) scheme. Specifically, for.

### 4.1 Effects of factors on metacomprehension monitoring accuracy and their interaction

The first research question asked whether pre-reading prediction and the encoding-retrieval interval affect metacomprehension monitoring accuracy from a cross-linguistic perspective, and whether these factors interact. Main effects analyses revealed a significant main effect of interval type (*b =* −0.27, *SE* *=* 0.05, *t =* −5.16, *p* < 0.001), and a significantly higher main effect of the non-interval condition (*M* = 0.64, *SD* = 0.69) than that of the interval condition (*M* = 0.05, *SD* = 0.92), suggesting that the non-interval condition significantly improved metacomprehension monitoring accuracy. The predictive main effect was significant (*b =* 0.44, *SE* *=* 0.21, *t =* 2.09, *p* < 0.05) and was significantly higher in the non-predictive condition (*M* = 0.37, *SD* = 0.84) than in the predictive condition (*M* = 0.33, *SD* = 0.88), suggesting that metacomprehension monitoring accuracy was higher in the non-predictive condition. The main effect of language type was not significant (*b* = 0.27, *p* > 0.05). Although the mean of the Chinese condition (*M* = 0.44, *SD* = 0.87) was higher than that of the English condition (*M* = 0.25, *SD* = 0.84), this difference did not reach a statistically significant level.

Interaction effects analyses revealed a significant interaction between language type and interval type either in the predictive condition (*b =* −0.57, *SE* *=* 0.26, *t =* −2.22, *p* < 0.05) and non-predictive condition (*b =* −0.79, *SE* *=* 0.33, *t =* −2.36, *p* = 0.02). The interaction effect of language type and predictive type was not significant (*b =* −0.36, *p* > 0.05), nor was the interaction effect of interval type and predictive type (*b =* −0.24, *p* > 0.05). The third-order interaction effect (language type × interval type × predictive) did not reach a significant level (*b =* −0.22, *p* > 0.05).

**Figure 1:**
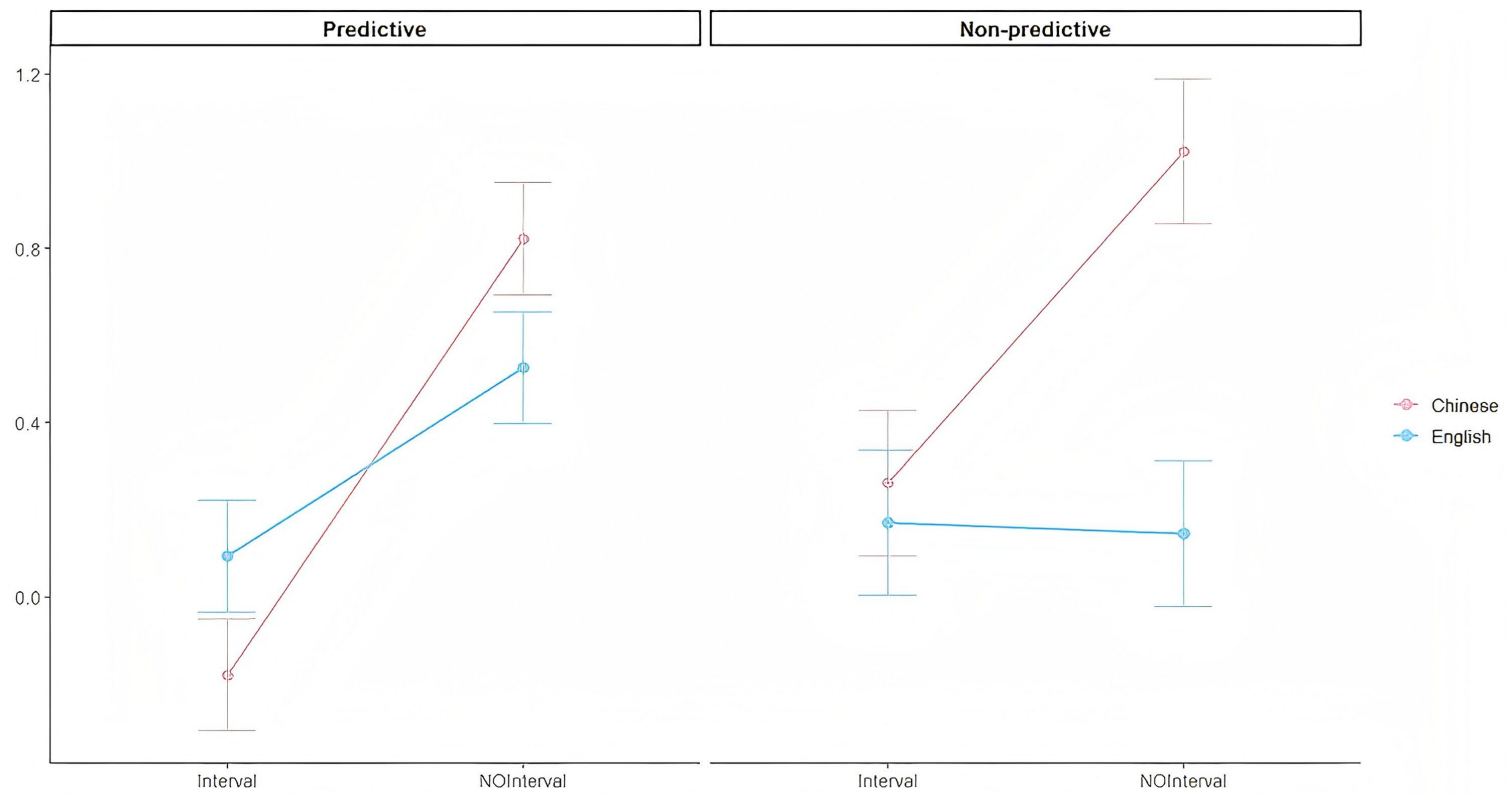
Interaction between language type and encoding-retrieval interval across prediction conditions.

To explore our theoretical hypotheses regarding the interplay between language type and interval type under different predictive conditions, we calculated three-factor marginal means and conducted simple effects analyses. The interaction patterns across these conditions are illustrated in Figure 1, which displays the three-way relationship among language type, interval type, and prediction condition. Specifically, the analysis revealed that the two-way interaction between language type and interval type was significant in both the predictive condition (*b* = −0.57, *SE* *=* 0.26, *t =* −2.22, *p* < 0.05) and the non-predictive condition (*b* = −0.79, *SE* *=* 0.33, *t =* −2.36, *p* < 0.05). In the predictive condition, (1) the non-interval condition was significantly better than the interval condition for readers reading in Chinese (*b* = −1.00, *SE* *=* 0.18, *t =* −5.51, *p* < 0.001), and the non-interval condition was also significantly better than the interval condition for readers reading in English (*b* = −0.43, *SE* *=* 0.18, *t =* −2.38, *p* < 0.05). (2) In the interval condition, the difference between English and Chinese was not significant (*b* = −0.27, *p* > 0.05), and in the non-interval condition, the difference between English and Chinese was also not significant (*b* = 0.30, *p* > 0.05). In the non-predictive condition: (1) for readers reading in Chinese, the non-interval condition was significantly better than the interval condition (*b* = −0.76, *SE* = 0.235, *t* = −3.234, *p* = 0.001) whereas for readers reading in English, the difference between the two interval conditions was not significant (*b* = 0.03, *p* > 0.05). (2) In the interval condition, the difference between Chinese and English was not significant (*b* = 0.09, *p* > 0.05). However, in the non-interval condition, Chinese reading condition was significantly better than the English reading condition (*b* = 0.88, *SE* *=* 0.24, *t =* 3.73, *p* < 0.001).

Taken together, these results indicate a robust effect of encoding-retrieval interval on metacomprehension monitoring accuracy across languages, with the interval effect interacting with language type such that the benefit of the non-interval condition was especially pronounced for readers reading in Chinese in some comparisons. The predicting manipulation overall decreased accuracy (higher accuracy in non-predictive condition), and no reliable evidence was found for two-way interactions involving predictive type or for a three-way interaction.

### 4.2 Cross-linguistic transfer effects: regression results and mediating effects

The second research question examined whether L1 (Chinese) metacomprehension monitoring accuracy transfers to L2 (English), and whether such transfer is mediated by target language proficiency. To investigate these questions, stratified forced-entry regression procedures were applied following Wen et al. (2005), and path analysis using structural equation modeling was conducted to test mediation.

#### 4.2.1 Regression results

First, the first level of regression analysis was conducted with native language metacomprehension monitoring accuracy and native language proficiency as predictor variables and foreign language metacomprehension monitoring accuracy as criterion variable. It was found that native language metacomprehension monitoring accuracy became a significant predictor of foreign language metacomprehension monitoring accuracy (*b =* 0.22, *SE* = 0.08, *t =* 2.58, *p* < 0.05), suggesting that native language metacomprehension monitoring accuracy may be subject to cross-linguistic transfer to the target language. Native language proficiency also showed a significant predictor (*b =* 0.03, *SE* = 0.01, *t =* 3.05, *p* < 0.001), indicating that native language proficiency has a positive effect on foreign language metacomprehension monitoring ability. Together, the two variables explained 12% of the total variance (*R*^2^ *=* 0.12, *p* < 0.001).

Next, foreign language proficiency was introduced as a predictor variable along with native language metacomprehension monitoring accuracy and native language proficiency for the second level of regression analyses while observing and comparing the changes in the *b*-value and *p*-value of native language metacomprehension monitoring accuracy. The results showed that native language metacomprehension monitoring accuracy maintained a significant predictive effect (*b =* 0.22, *SE* = 0.01, *t =* 2.61, *p* < 0.05), a result that further confirms the positive effect of native language metacomprehension monitoring accuracy on foreign language metacomprehension monitoring ability. The predictive effect of native language proficiency remained significant (*b =* 0.03, *t =* 3.08, *p* < 0.001), a result that further confirms the positive effect of native language proficiency on foreign language metacomprehension monitoring ability. In contrast, the predictive effect of foreign language proficiency was not significant (*b =* 0.01, *p > 0.*05), which suggests that foreign language proficiency did not significantly influence foreign language metacomprehension monitoring ability, with the three variables together explaining 12% of the total variance (*R*^2^ *=* 0.12, *p* < 0.001).

#### 4.2.2 Mediating effects

**Figure 2:**
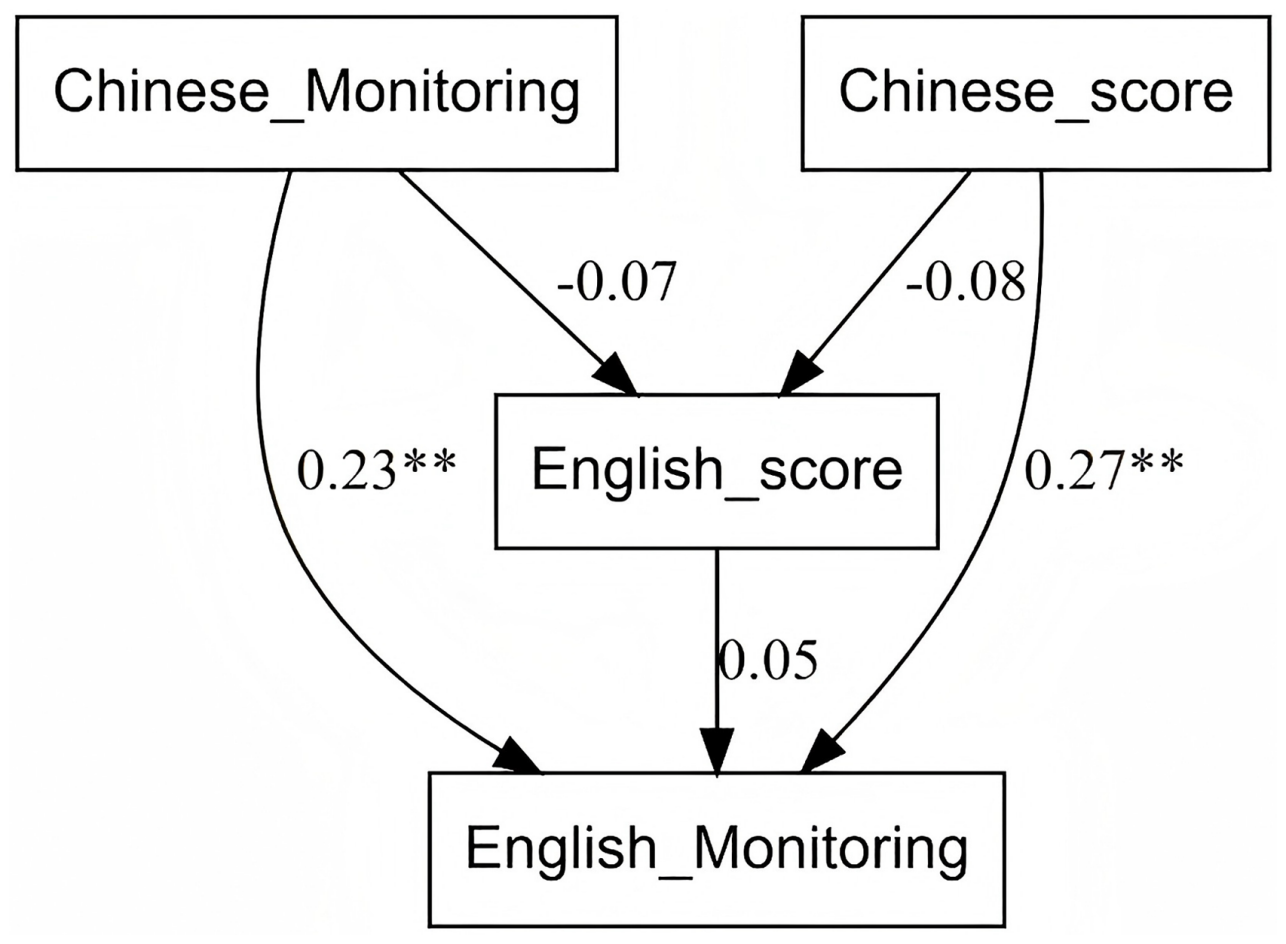
Mediating effect of L2 proficiency on cross-linguistic transfer of monitoring.

To further test whether L2 proficiency mediates the transfer from L1 monitoring to L2 monitoring, path analysis was performed (Figure 2).

1. Direct effects in path relationships

The direct effect of native language metacomprehension monitoring accuracy on foreign language metacomprehension monitoring accuracy was significant (*b =* 0.22, *p* < 0.001), indicating that native language metacomprehension monitoring ability can directly and positively influence foreign language metacomprehension monitoring accuracy. The direct effect of native language proficiency on foreign language metacomprehension monitoring accuracy was significant (*b =* 0.03, *p* < 0.001), suggesting that native language proficiency can also directly and positively influence foreign language metacomprehension monitoring accuracy. The direct effect of foreign language proficiency on foreign language metacomprehension monitoring accuracy was not significant (*b =* 0.01, *p* > 0.05), suggesting that foreign language proficiency itself has a limited direct effect on foreign language metacomprehension monitoring accuracy.

1. Mediating paths in path relationships

The mediation path of native language metacomprehension monitoring accuracy on L2 proficiency was not significant (*b =* −0.58, *p* > 0.05), indicating that L1 metacomprehension monitoring accuracy does not have a significant direct effect on L2 proficiency. The mediation path of L1 proficiency on foreign language proficiency was not significant (*b =* −0.08, *p* > 0.05), indicating that L1 proficiency has no significant direct effect on foreign language proficiency.

Comparing the results of the before and after regression analyses, when native metacomprehension monitoring accuracy, L2, and L1 proficiency were included in the regression model at the same time, the predictive effects of native metacomprehension monitoring accuracy and L1 proficiency remained significant, and there was a slight increase in *b* value for native metacomprehension monitoring accuracy and a slight increase in *t* value for L1 proficiency, but the predictive effect of L2 proficiency was not significant (*b =* 0.01, *p* > 0.05). This result is different from expectations and suggests that L2 proficiency may not play a significant mediating role in the transfer of L1 metacomprehension monitoring accuracy to the L2. Path analyses further confirmed this finding, showing that both L1 metacomprehension monitoring accuracy (*b =* 0.22, *p* < 0.001) and L1 proficiency (*b =* 0.03, *p* < 0.001) had a significant direct effect on L2 metacomprehension monitoring accuracy, whereas the mediating path through L2 proficiency was not significant. Mediating effect of L2 proficiency on cross-linguistic transfer of monitoring is shown in Figure 2.

## 5 Discussion

### 5.1 Effects of pre-reading prediction and encoding-retrieval interval on metacomprehension monitoring accuracy

The first research question examined whether pre-reading prediction and encoding-retrieval interval influence metacomprehension monitoring accuracy, and whether these factors interact. Consistent with the transfer-appropriate monitoring hypothesis (Koriat and Ma’ayan, 2005; Rawson and Dunlosky, 2002; Thiede et al., 2009), the non-interval condition yielded significantly higher monitoring accuracy than the interval condition. Specifically, immediate judgments allowed direct access to working memory content, supporting accurate situation model construction (Anderson and Thiede, 2008; Thiede et al., 2009).

In contrast, a significant main effect of pre-reading prediction emerged in the opposite direction. Monitoring accuracy was higher in the non-predictive condition. This can be explained by the nature of the expository texts used here. These texts were low in coherence and contained domain-specific content for which participants lacked relevant prior knowledge (Graesser et al., 2011; Weaver and Bryant, 1995). Under such conditions, generating predictions likely produced inaccurate expectations that interfered with comprehension, thereby reducing monitoring accuracy.

Moreover, the interaction between language type and interval type was also significant. Specifically, when reading in Chinese, the non-interval advantage was consistent regardless of prediction instruction. In contrast, when reading in English, the non-interval advantage appeared only under predictive instruction; in the non-predictive non-interval condition, performance was significantly worse. This pattern reflects differences in processing automaticity between two languages (DeKeyser, 2007; Segalowitz and Hulstijn, 2005). Reading in Chinese benefits from highly automatized lexical access, enabling efficient situation model construction. However, reading in English involves more effortful processing that consumes working memory resources and reduces monitoring accuracy. Therefore, predictive instruction appears to compensate for this burden by directing attention to diagnostic cues. This interpretation is supported by Bagri and Abrar (2024) who found that students of high verbal working memory showed greater metacomprehension and underconfidence after strategy instruction. But students of low verbal working memory exhibited overconfidence and shorter reading time. This pattern is analogous to the effects of prediction for the more demanding English reading condition in our study.

Besides, the observation that predictive instruction was beneficial for English but not for Chinese readers aligns with the goal-congruence effect reported by Mengelkamp et al. (2025). In their research, performance on inferential questions was highest when reading goals matched the text genre. For the current study, prediction may have served as some kind of study goal that was consistent with reading in a more demanding L2 while being redundant or possibly deistracting when reading in L1.

Finally, future research could directly manipulate or measure prior knowledge accessibility at the moment of prediction (e.g., using think-aloud protocols or domain training) and include verbal working memory measures to better understand the cognitive mechanisms underlying cross-linguistic differences.

### 5.2 Cross-linguistic transfer and the mediating role of L2 proficiency

The second research question asked whether metacomprehension monitoring ability transfers from L1 to L2, and whether L2 proficiency mediates this transfer. As shown by regression and path analyses, L1 monitoring accuracy significantly predicted L2 monitoring accuracy, providing direct evidence for cross-linguistic transfer. This finding supports the Reading Universal Hypothesis (Coady, 1979) that posits that reading skills including metacognitive monitoring are domain-general and transfer across languages regardless of specific linguistic knowledge. In contrast, the Linguistic Threshold Hypothesis (Pichette and Segalowitz, 2003) would predict that transfer requires a minimum level of L2 proficiency.

However, L2 proficiency did not mediate this transfer. The results showed that L2 proficiency did not significantly mediate the transfer; instead, the direct effect of L1 monitoring on L2 monitoring remained robust after controlling for L2 proficiency. Two complementary explanations account for this finding. First, metacomprehension monitoring may draw on domain-general metacognitive skills that transfer independently of language-specific competencies. Skills such as self-evaluation, strategy regulation, and comprehension assessment are not tied to any particular language. This interpretation is strongly supported by recent research on transfer of metacognitive regulation. Wirth et al. (2025) demonstrated that learners can transfer metacognitive regulation skills acquired in combination with one cognitive learning strategy to a different resource management strategy. This transfer effect provides a direct theoretical mechanism for cross-linguistic transfer, that is, if metacognitive regulation can transfer across qualitatively different types of learning strategies, it can also transfer across different languages. Similarly, Proust et al. (2025) found that a simple procedural intervention, i.e., routinely asking students to predict and evaluate their own performance before comparing their judgments with outcomes, namely improvement in predictive metacognitive sensitivity. This indicates that metacomprehension monitoring can be trained procedurally without being bound to a specific language.

Second, the sample comprised first-year English majors with relatively high and clustered L2 proficiency, which may have reduced statistical sensitivity to detect a threshold effect. Our participants were first-year majors with relatively high and clustered L2 proficiency. Furthermore, the use of college entrance examination scores as a covariate. While these scores are standardized, does not capture finer grained aspects of proficiency such as reading-specific skills or vocabulary depth. Thus, the null mediating effect may reflect both restricted variability and the genuine prominence of domain-general skills. This limitation corresponds to the findings of Seban et al. (2025) on-line (judgment-based) measures of metacognition that correlates closely with performance while off-line (self-report) measures do not. As a result, the absence of mediating effect may reflect both restricted variability and the genuine prominence of domain-general metacognitive skills.

Moreover, the absence of mediation also aligns with findings in other research. Witherby et al. (2023) found that prior knowledge had negative effects on predicting Judgement of Learning (JOL) accuracy for new domain-relevant items because learners of high knowledge could often remember items they might forget. In the current study, high L2 proficiency may not guarantee better monitoring accuracy and may lead to a mismatch between prediction and performance similar to the finding of Witherby et al. This further supports the conclusion that domain-general metacognitive skills and not the language proficiency drive the transfer effect.

Nevertheless, the homogeneity of the sample also limits generalizability, namely, the observed interval and prediction effects may be characteristic of relatively proficient university L2 learners and may not extend to less proficient learners, younger students, or other educational contexts. Therefore, future research should replicate the design with more diverse samples across proficiency levels, age groups, and institutional settings.

In conclusion, the results answer the second research question affirmatively in that there is clear cross-linguistic transfer of metacomprehension monitoring ability between Chinese (L1) and English (L2). However, this transfer is not mediated by L2 proficiency, indicating that the mechanism is primarily domain-general rather than language-specific. This finding is consistent with the broader literature on transfer of metacognitive skills (Proust et al., 2025; Wirth et al., 2025) and with evidence that prior knowledge can paradoxically reduce monitoring accuracy (Witherby et al., 2023).

## 6 Conclusion

In summary, this study yielded three main findings. First, immediate (non-interval) access to comprehension cues significantly improved metacomprehension monitoring accuracy. Second, pre-reading prediction reduced monitoring accuracy for unfamiliar expository texts, likely because generating predictions without sufficient prior knowledge interfered with comprehension. Third, L1 metacomprehension monitoring ability transferred to L2 reading, but this transfer was not mediated by L2 proficiency, supporting the Reading Universal Hypothesis and suggesting that domain-general metacognitive skills play a primary role.

These findings advance understanding of both language-specific and language-general mechanisms in metacognitive comprehension and provide practical guidance for integrating metacognitive instruction into language education. However, the conclusions should be interpreted with caution due to the homogeneity of the sample in that first-year English majors from a single university with relatively high and clustered L2 proficiency. This restricted range may have reduced the sensitivity to detect a potential L2 proficiency threshold and limits generalizability to less proficient learners, other educational levels, or different institutional contexts. Future research should therefore replicate the design with more diverse samples across proficiency levels, age groups, and settings, and consider including verbal working memory measures to further elucidate the cognitive mechanisms underlying cross-linguistic differences.

## Data Availability

The raw data supporting the conclusions of this article will be made available by the authors, without undue reservation.

## Appendix I

Example:

Article title: “*The Uniform Paradox: Professionalism vs Individuality*.”

Topic prediction questions:

“Based on the title of the article, which of the following topics do you think this article is most likely to discuss?”

- The history of uniforms in the United States.
- The conflict between professionalism and individuality in wearing uniforms.
- The economic benefits of wearing uniforms.
- The psychological effects of uniforms on employees.

## Appendix II

Example:

Article title: “*The Uniform Paradox: Professionalism vs Individuality*.”

Structural content prediction questions:

“Americans are proud of their variety and individuality, yet they love and respect few things more than a uniform, whether it is the uniform of an elevator operator or the uniform of a five-star general. Why are uniforms so popular in the United States.”

Among the arguments for uniforms, one of the first is that, in the eyes of most people, they look more professional than civilian clothes.

Based on the title and part of the article, what will the article most likely discuss next?

- The psychological effects of uniforms on people’s behavior.
- The practical benefits of wearing uniforms.
- The history of uniforms in different professions.
- The economic impact of uniforms on businesses.

## Appendix III: Examples of detail and inference questions

Example:

Text excerpt (from the passage “The Office Politics Game: How to Play and Win”):

*A question on the topic*:

What is the potential topic of the article based on the title? ().

- How to work hard to get a promotion at work.
- The rules of office politics and how to succeed in it.
- How to build good relationships with coworkers.
- How to avoid conflicts in the workplace.

“You may be all these things at the office, and more. But when it comes to getting ahead, experts say, the ABCs of business should include a P, for politics, as in office politics.

Dale Carnegie suggested as much more than 50 years ago: Hard work alone does not ensure career advancement. You have to be able to sell yourself and your ideas, both publicly and behind the scenes. Yet, despite the obvious rewards of engaging in office politics—a better job, a raise, praise—many people are still unable—or unwilling—to play the game.”

Based on the title and the first two paragraphs, what will the article most likely discuss next? ().

- Why hard work is not enough for career success.
- Examples of successful people who used office politics.
- How to balance work and personal life.
- The history of office politics.

## Appendix IV: Metacomprehension rating scale example

Example:

Please rate your comprehension of each text on a 1–5 scale (1 = did not understand at all, 5 = understood completely; the closer to 1, the less you understood; the closer to 5, the better you understood). Please mark a tick (√) in the corresponding number.

Text 112345

……

## References

[ref1] Ackerman R. Binah-Pollak A. Lauterman T. (2023). Metacognitive effort regulation across cultures. J. Intelligence 11:171. doi: 10.3390/jintelligence11090171, 37754900 PMC10532471

[ref2] Anderson M. C. M. Thiede K. W. (2008). Why do delayed summaries improve metacomprehension accuracy? Acta Psychol. 128, 110–118. doi: 10.1016/j.actpsy.2007.10.006, 18070613

[ref3] Bagri G. Abrar S. (2024). Strategy instruction and working memory on meta-comprehension, confidence, and test prediction accuracy for expository text in students. Cogent Educ. 11:2372189. doi: 10.1080/2331186X.2024.2372189

[ref5] Bates D. Mächler M. Bolker B. Walker S. (2015). Fitting linear mixed-effects models using lme4. J. Stat. Softw. 67, 1–48. doi: 10.18637/jss.v067.i01

[ref6] Belsley D. A. Kuh E. Welsch R. E. (1980). Regression Diagnostics: Identifying Influential data and Sources of Collinearity. Hoboken: John Wiley & Sons.

[ref7] Brown A. L. (1982). Inducing Strategic Learning from Texts by means of Informed, Self-Control Training (Technical Report No. 262). Champaign: University of Illinois at Urbana-Champaign, Center for the Study of Reading.

[ref8] Cain K. Oakhill J. V. (1999). Inference making and its relation to comprehension failure. Read. Writ. 11, 489–503. doi: 10.1023/A:1008084120205

[ref9] Chen Q. S. Chang R. (2009). Why are readers’ metacomprehension monitoring judgments not accurate? Adv. Psychol. Sci. 17, 706–713. doi: 10.3724/SP.J.1041.2008.00961

[ref10] Chen Q. S. Li L. (2008). Rating comprehension and predicting performance: clarifying two forms of metacomprehension monitoring. Acta Psychol. Sin. 40, 961–968. doi: 10.3724/SP.J.1041.2008.00961

[ref11] Chen Q. S. Wen Z. L. (2010). Metacognition and effective learning: principles and conditions. J. East China Normal Univ. (Educ. Sci.) 1, 55–61.

[ref12] Coady J. (1979). “A psycholinguistic model of the ESL reader,” in Reading in a Second Language, eds. Mackay R. Barkman B. Jordan R. R. (Mayfield: Newbury House), 5–12.

[ref13] DeKeyser R. (2007). “Skill acquisition theory,” in Theories in second Language Acquisition: An Introduction, eds. VanPatten B. Williams J. (Mahwah: Lawrence Erlbaum Associates), 97–113.

[ref14] Duan S. Q. (2004). A brief discussion of prediction and its methods in reading comprehension. Teach. Manag. 10:44.

[ref15] Dunlosky J. Lipko A. (2007). Metacomprehension: a brief history and how to improve its accuracy. Curr. Dir. Psychol. Sci. 16, 228–232. doi: 10.1111/j.1467-8721.2007.00509.x

[ref17] Dunlosky J. Rawson K. A. Hacker D. (2002). “Metacomprehension of science text: investigating the levels-of-disruption hypothesis,” in The Psychology of Science text Comprehension, eds. Otero J. Graesser A. (Mahwah: Lawrence Erlbaum Associates), 255–280.

[ref19] Flavell J. H. (1979). Metacognition and cognitive monitoring: a new area of cognitive–developmental inquiry. Am. Psychol. 34, 906–911. doi: 10.1037/0003-066X.34.10.906

[ref21] Goodman K. S. (1967). Reading: a psycholinguistic guessing game. J. Read. Spec. 6, 126–135. doi: 10.1080/19388076709556976

[ref22] Gordon C. J. Pearson P. D. (1983). The Effects of Instruction in Metacomprehension and Inferencing on Children’s Comprehension Abilities (Technical Report No. 277). Champaign: University of Illinois at Urbana-Champaign, Center for the Study of Reading.

[ref23] Graesser A. C. McNamara D. S. Kulikowich J. M. (2011). Coh-metrix: providing multilevel analyses of text characteristics. Educ. Res. 40, 223–234. doi: 10.3102/0013189X11413260

[ref24] Griffin T. D. Wiley J. Thiede K. W. (2008). Individual differences, rereading, and self-explanation: concurrent processing and cue validity as constraints on metacomprehension accuracy. Mem. Cogn. 36, 93–103. doi: 10.3758/MC.36.1.93, 18323066

[ref28] Kintsch W. (1988). The use of knowledge in discourse processing: a construction-integration model. Psychol. Rev. 95, 163–182. doi: 10.1037/0033-295X.95.2.163, 3375398

[ref29] Koriat A. (1997). Monitoring one’s own knowledge during study: a cue-utilization approach to judgments of learning. J. Exp. Psychol. Gen. 126, 349–370. doi: 10.1037/0096-3445.126.4.349

[ref30] Koriat A. Ma’ayan H. (2005). The effects of encoding fluency and retrieval fluency on judgments of learning. J. Mem. Lang. 52, 478–492. doi: 10.1016/j.jml.2005.01.001

[ref32] Li M. Xu L. (2022). An analysis of the correlation between college entrance examination scores and university performance. J. Yunnan Minzu Univ. (Nat. Sci. Edn.) 31, 360–365.

[ref33] Lin L. M. Moore D. Zabrucky K. M. (2000). Metacomprehension knowledge and comprehension of expository and narrative texts among younger and older adults. Educ. Gerontol. 26, 737–749.

[ref34] Lin L. M. Zabrucky K. M. (1998). Calibration of comprehension: research and implications for education and instruction. Contemp. Educ. Psychol. 23, 345–391. doi: 10.1006/ceps.1998.0972, 9769183

[ref35] Liu H. Zhang P. Pan J. (2018). How to make the results of academic evaluation more valid: research on adjustment model based on latent variable. J. East China Normal Univ. (Educ. Sci.) 36, 87–98. doi: 10.16382/j.cnki.1000-5560.2018.03.009

[ref37] Maki R. H. Shields M. Wheeler A. E. Zacchilli T. L. (2005). Individual differences in absolute and relative metacomprehension accuracy. J. Educ. Psychol. 97, 723–731. doi: 10.1037/0022-0663.97.4.723

[ref38] Matlin M. W. (2005). Cognition. 6th Edn Hoboken: John Wiley & Sons.

[ref39] Matsumoto Y. (2011). Keep your students awake: using prediction in a reading class. Lang. Teach. 35, 27–29. doi: 10.37546/JALTTLT35.3

[ref40] McNamara D. S. (2004). SERT: self-explanation reading training. Discourse Process. 38, 1–30. doi: 10.1207/s15326950dp3801_1

[ref41] Mengelkamp C. Golke S. Appel M. (2025). Effects of reading goal instructions on the comprehension and metacomprehension of informative narratives. Appl. Cogn. Psychol. 39:e70036. doi: 10.1002/acp.70036

[ref36] Morrison L. (2004). Comprehension monitoring in first and second language reading. Can. Mod. Lang. Rev. 61, 77–106. doi: 10.3138/cmlr.61.1.77

[ref43] Moss J. Schunn C. D. (2015). Comprehension through explanation as the interaction of the brain’s coherence and cognitive control networks. Front. Hum. Neurosci. 9, 562. doi: 10.3389/fnhum.2015.00562 26557066 PMC4615809

[ref44] Murphy J. D. Klin C. M. Myers J. L. (1993). Forward inferences in narrative text. J. Mem. Lang. 32, 464–473. doi: 10.1006/jmla.1993.1025

[ref45] Nelson T. O. Narens L. Dunlosky J. (2004). A revised methodology for research on metamemory: pre-judgment recall and monitoring (PRAM). Psychol. Methods 9, 53–69. doi: 10.1037/1082-989X.9.1.53, 15053719

[ref46] Nuttall C. (2002). Teaching reading Skills in a Foreign Language. Shanghai: Shanghai Foreign Language Education Press.

[ref47] Pan W. G. (2005). Outline of Chinese-English Contrastive Studies. Beijing: Beijing Language and Culture University Press.

[ref48] Pichette F. Segalowitz N. (2003). Impact of maintaining L1 reading skills on L2 reading skill development in adults: evidence from speakers of SerboCroatian learning French. Mod. Lang. J. 87, 391–403. doi: 10.1111/1540-4781.00197

[ref49] Prinz A. Golke S. Wittwer J. (2020). How accurately can learners discriminate their comprehension of texts? A comprehensive meta-analysis on relative metacomprehension accuracy and influencing factors. Educ. Res. Rev. 31:100358. doi: 10.1016/j.edurev.2020.100358

[ref50] Proust J. Guilleray F. Serveau V. Goupil L. (2025). Can metacognitive monitoring be trained procedurally in the classroom? Metacogn. Learn. 20:13. doi: 10.1007/s11409-025-09418-0

[ref51] R Core Team (2022). R: A Language and Environment for Statistical Computing. Vienna: R Foundation for Statistical Computing.

[ref52] Rawson K. A. Dunlosky J. (2002). Are performance predictions for text based on ease of processing? J. Exp. Psychol. Learn. Mem. Cogn. 28, 69–80. doi: 10.1037/0278-7393.28.1.69, 11827088

[ref53] Rawson K. A. Kintsch W. (2005). Rereading effects depend on time of test. J. Educ. Psychol. 97, 70–80. doi: 10.1037/0022-0663.97.1.70

[ref54] Rumelhart D. E. (1980). “Schemata: the building blocks of cognition,” in Theoretical Issues in reading Comprehension, eds. Spiro R. J. Bruce B. C. Brewer W. F. (Mahwah: Lawrence Erlbaum Associates), 33–58.

[ref55] Seban P. Sikl R. Urban K. (2025). Relationship between monitoring judgments and self-report measures of metacognition in educational research. Int. J. Educ. Res. 131:102578. doi: 10.1016/j.ijer.2025.102578

[ref56] Segalowitz N. Hulstijn J. (2005). “Automaticity in bilingualism and second language learning,” in Handbook of Bilingualism: Psycholinguistic Approaches, eds. Kroll J. F. de Groot A. M. B. (Oxford: Oxford University Press), 371–388.

[ref57] Shi C. (2025). A case study of National Matriculation English Test validity in Predicting Students’ English performance in college. SAGE Open 15:21582440251331196. doi: 10.1177/21582440251331196

[ref58] Soto C. de Gutierrez Blume A. P. Jacovina M. McNamara D. Benson N. Riffo B. (2019). Reading comprehension and metacognition: The importance of inferential skills. Cogent Educ. 6:1565067. doi: 10.1080/2331186X.2019.1565067

[ref59] Thiede K. W. Anderson M. C. M. Therriault D. (2003). Accuracy of metacognitive monitoring affects learning of texts. J. Educ. Psychol. 95, 66–73. doi: 10.1037/0022-0663.95.1.66

[ref60] Thiede K. W. Dunlosky J. Griffin T. D. Wiley J. (2005). Understanding the delayed-keyword effect on metacomprehension accuracy. J. Exp. Psychol. Learn. Mem. Cogn. 31, 1267–1280. doi: 10.1037/0278-7393.31.6.1267, 16393046

[ref61] Thiede K. W. Griffin T. D. Wiley J. Redford J. S. (2009). “Metacognitive monitoring during and after reading,” in Handbook of Metacognition in Education, eds. Dunlosky J. Graesser A. Hacker J. (Cambridge: Routledge), 85–106.

[ref62] Wang D. Li L. (2014). The application of prediction strategies in senior high school English reading instruction. Teach. Month. (Second. Edn. Teach. Ref.) 10, 21–23.

[ref63] Weaver C. A. Bryant D. S. (1995). Monitoring of comprehension: the role of text difficulty in metamemory for narrative and expository text. Mem. Cogn. 23, 12–22. doi: 10.3758/bf03210553, 7885261

[ref64] Wen Z. L. Hou J. T. Zhang L. (2005). Comparison and application of moderation and mediation effects. Acta Psychol. Sin. 2, 268–274.

[ref66] Wirth J. Weber-Reuter X.-L. Schuster C. Fleischer J. Leutner D. Stebner F. (2025). Far transfer of metacognitive regulation: from cognitive learning strategy use to mental effort regulation. Educ. Psychol. Rev. 37:7. doi: 10.1007/s10648-024-09983-x

[ref67] Witherby A. E. Carpenter S. K. Smith A. M. (2023). Exploring the relationship between prior knowledge and metacognitive monitoring accuracy. Metacogn. Learn. 18, 591–621. doi: 10.1007/s11409-023-09344-z

[ref69] Yan R. Li T. Li S. Yu H. X. (2015). Effects of clue encoding–retrieval time intervals on the accuracy of metacomprehension monitoring in foreign-language reading. J. Beijing Int. Stud. Univ. 37, 1–6.

[ref70] Yang D. Y. Qin X. F. (2007). Survey and analysis of English reading strategies of freshman English majors. Education and Vocation 8, 184–186.

[ref71] Yu H. (2006). Reading prediction in multimedia contexts. J. Huazhong Agric. Univ. (Soc. Sci.) 5, 132–136.

[ref72] Zhao X. Zhao J. Guo X. Wu Y. (2022). A study on the reliability and validity of Gaokao based on correlation analysis. J. Chin: Exam, 30, 37–43. doi: 10.19360/j.cnki.11-3303/g4.2022.03.006

